# Endothelial-specific deficiency of megalin in the brain protects mice against high-fat diet challenge

**DOI:** 10.1186/s12974-020-1702-2

**Published:** 2020-01-14

**Authors:** Fernando Bartolome, Desiree Antequera, Macarena de la Cueva, Marcos Rubio-Fernandez, Nerea Castro, Consuelo Pascual, Antoni Camins, Eva Carro

**Affiliations:** 1 Neurodegenerative Disorders Group, Instituto de Investigacion Hospital 12 de Octubre (i+12), Avda de Cordoba s/n, 28041 Madrid, Spain; 2 Network Center for Biomedical Research in Neurodegenerative Diseases, CIBERNED, Madrid, Spain; 3 Unitat de Farmacologia i Farmacognosia, Facultat de Farmacia, Institut de Biomedicina de la UB (IBUB), Universitat de Barcelona, Barcelona, Spain

**Keywords:** Megalin, Leptin, Obesity, High-fat diet, Mitochondrial biogenesis

## Abstract

**Background:**

The increasing risk of obesity and diabetes among other metabolic disorders are the consequence of shifts in dietary patterns with high caloric-content food intake. We previously reported that megalin regulates energy homeostasis using blood-brain barrier (BBB) endothelial megalin-deficient (EMD) mice, since these animals developed obesity and metabolic syndrome upon normal chow diet administration. Obesity in mid-life appears to be related to greater dementia risk and represents an increasing global health issue. We demonstrated that EMD phenotype induced impaired learning ability and recognition memory, neurodegeneration, neuroinflammation, reduced neurogenesis, and mitochondrial deregulation associated with higher mitochondrial mass in cortical tissues.

**Methods:**

EMD mice were subjected to normal chow and high-fat diet (HFD) for 14 weeks and metabolic changes were evaluated.

**Results:**

Surprisingly, BBB megalin deficiency protected against HFD-induced obesity improving glucose tolerance and preventing hepatic steatosis. Compared to wild type (wt), the brain cortex in EMD mice showed increased levels of the mitochondrial biogenesis regulator, peroxisome proliferator-activated receptor γ coactivator-1α (PGC-1α), and uncoupling protein 2 (UCP2), a thermogenic protein involved in the regulation of energy metabolism. This agreed with the previously found increased mitochondrial mass in the transgenic mice. Upon HFD challenge, we demonstrated these two proteins were found elevated in wt mice but reported no changes over the already increased levels in EMD animals.

**Conclusion:**

We propose a protective role for megalin on diet-induce obesity, suggesting this could be related to metabolic disturbances found in dementia through brain endocrine system communications.

## Background

Urban lifestyle conducts changes in human eating habits, in which intake of high caloric content foods come along with reduced physical activity. This context is putatively associated with many epidemic chronic diseases that have emerged in relatively recent times. For example, obesity has been found to increase in developed countries where high-calorie food intake is a major cause of this global health problem. Food intake and energy expenditure balance modulate body weight, and this is regulated mainly through hormone leptin [1]. Leptin is internalized by the multiligand endocytic receptor megalin, also known as low-density lipoprotein receptor-related protein 2 (LRP-2) or glycoprotein 330, the largest member of the low-density lipoprotein receptor (LDLR) family [2, 3]. Megalin is expressed in several absorptive epithelial cells, and in the central nervous system (CNS) mainly in the blood-brain barrier (BBB). Because megalin binds and internalizes leptin and insulin [4–6], and megalin is expressed exclusively in brain endothelial cells, we previously set out to specifically delete the endothelial megalin in C57/BL6 mice using the Cre/loxP system (EMD mice) [7, 8] to explore the metabolic impact of BBB megalin deletion [9]. These EMD mice developed neurodegeneration and impaired learning and memory abilities, similar to symptoms described in AD [9]. Also, we reported this mouse model displayed obesity and metabolic syndrome, mediated by leptin signaling disruption in the hypothalamus, upon normal chow diet administration [10]. Hence, we consider this model gives the opportunity to explore and understand several overlapping and common mechanisms, including mitochondrial dysfunction, that is observed in these disorders.

Obesity in mid-life appears to be related to greater dementia risk and there are several studies reporting this connection [11–15]. This is consistent with the observed higher Alzheimer’s disease (AD) incidence in world regions associated with high risk of obesity, sedentarism, diabetes, hypertension, dyslipidemia, and metabolic syndrome [16, 17]. A number of adverse neuronal effects have been observed under obese conditions [18]. Higher dietary fat intake has been associated with increased AD risk [19]. Also, using common AD mouse models, it has been found diet-induced obesity accelerates AD-related pathology [20–24]. Paradoxically, it is known that AD subjects show a hypermetabolic state accompanied by increased energy expenditure. This makes AD patients undergo a significant weight loss even when they have increased food intake [25]. These features have been observed still in patients with the preclinical condition of mild cognitive impairment (MCI) [26, 27]. In our EMD mouse model, we firstly demonstrated that BBB megalin deletion induced impaired learning ability and recognition memory, and neurodegeneration, similar to symptoms described in AD [9]. More recently, we also showed these mice displayed neuroinflammation, reduced neurogenesis and mitochondrial deregulation associated with higher mitochondrial mass in cortical tissues [10].

In the present study, we investigated the effects of HFD challenge in EMD mice compared to wt animals. We found that BBB megalin deletion preserved HFD-induced metabolic alterations and obesity. We consider it important to fully characterize EMD mice as this model constitutes a valuable obesity model, linking obesity and neurodegeneration as previously was already demonstrated that megalin deletion in brain endothelial cells could be a novel mechanism to promote neurodegeneration and obesity. Our mice model would help to understand the molecular mechanisms that may link obesity and dementia as obesity is proposed as a putative risk factor for AD.

## Methods

### Animals and diets

**Fig. 1:**
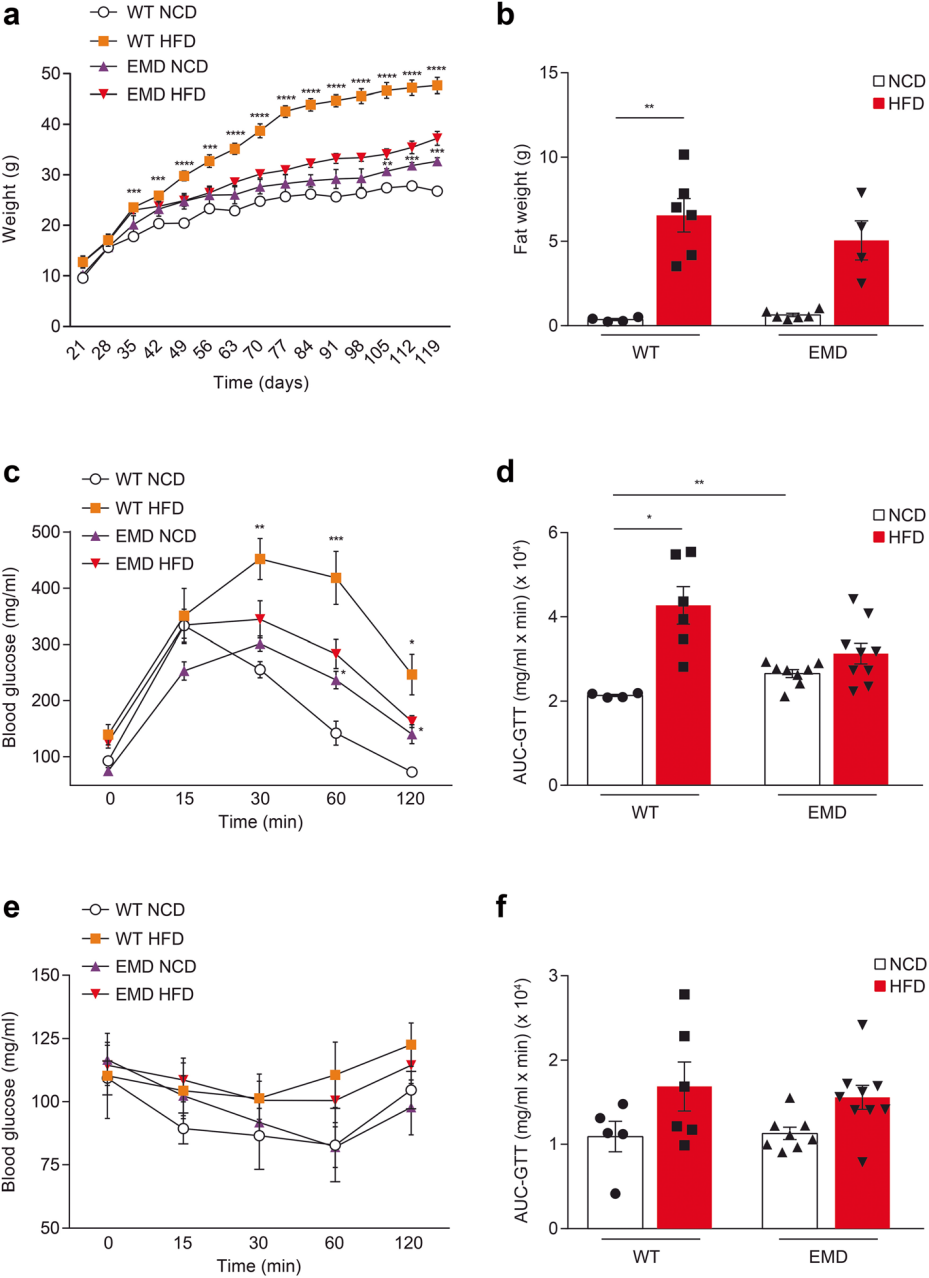
EMD mice display a significant reduction in HFD-induced obesity phenotype. Wt and EMD male mice were fed with NCD or HFD for 14 weeks. **a** Under NCD, bodyweight gain during 14-week feeding was significantly increased in EMD mice compared to wt. HFD administration significantly increased body weight in both mice groups but this increase was much more significant in wt group (*n* = 9, in all animal groups). **b** Fat weight in NCD-fed or HFD-fed mice at week 14. **c** Glucose tolerance test in wt and EMD mice fed with NCD or HFD for 14 weeks in the 16-h-fasted state (wt NCD, *n* = 5; wt HFD, *n* = 6; EMD NCD, *n* = 8; EMD HFD *n* = 9). **d** Scatter plots with bars represent the area under the glucose curve from the glucose tolerance test. **e** Insulin tolerance test in wt and EMD mice fed with NCD or HFD for 14 weeks in the 6-h-fasted state (wt NCD, *n* = 5; wt HFD, *n* = 6; EMD NCD, *n* = 8; EMD HFD *n* = 9). **f** Scatter plots with bars represent the area under the glucose curve from the insulin tolerance test. All data are presented as the mean ± SEM. Statistical significance in a, c and e is based on multivariate ANOVA analysis followed by Games-Howell post hoc test. Statistical significance in **b**, **d**, and **f** was based on one-way ANOVA followed by Games-Howell’s (**b** and **d**) or Tukey’s (**f**) post hoc test. *P < 0.05, **P < 0.01, ***P < 0.001, ****P < 0.0001

Male EMD mice were generated using the Cre/loxP system under the control of the Tie2 as previously reported [10]. Wild-type (wt) littermates (i.e., Tie2-Cre − mice) were used as controls (megalin flox/flox). EMD and control mice were housed on a 12 h light/12 h dark schedule. At the age of 3 weeks, male EMD and WT mice (16–18 per group) were provided a normal chow diet (NCD, 10% fat in kilocalories) or high-fat diet (HFD) containing 60% fat (Harlan Teklad, USA) ad libitum for 14 weeks. Bodyweight was monitored weekly throughout the study. At the end of experiments, animals were anesthetized with isoflurane, blood was drawn, and perfused transcardially with saline buffer or 4% paraformaldehyde in 0.1 M phosphate buffer (PB, pH 7.4) for biochemical and immunohistochemical analysis, respectively. Then, the brain, liver, and adipose tissue were collected for further processing and stored at − 80 °C until analysis. The liver and adipose tissue were previously weighed. Visceral fat was collected and weighed prior to − 80 °C storage, and average obtained weights per group are shown expressed in grams (Fig. 1b). All animals were handled and cared for according to the Council Directive 2010/63/UE of 22 September 2010 (animal experiment license number: CEI 97–1778–A291).

### Metabolic studies

Glucose tolerance test was assessed prior to sacrifice. After 14 weeks under the diets, a glucose tolerance test was performed. Briefly, mice were fasted for 16 h before receiving intraperitoneal (ip) administration of 2 g/kg body weight of glucose in saline (0.9% NaCl). Blood samples of conscious mice were taken from the tail vein at 0, 15, 30, 60, and 120 min after glucose loading, and the blood glucose levels were determined. Blood glucose measurements were performed using a glucometer (Accu-Chek, Roche, Mannheim, Germany).

The insulin-tolerance test was performed with male mice after 6 h fast. The animals were ip injected with 0.75 UI/kg body weight of insulin (Actrapid, Novo Nordisk Pharma, Bagsvaerd, Denmark). Blood glucose measurements were performed with the glucometer before the injection and at 15, 30, 60, and 120 min after the injection. Serum triglycerides (TG), total cholesterol, HDL cholesterol, non-esterified fatty acids (NEFA), leptin, and insulin were determined using commercial kits from Abcam (Abcam, Cambridge, UK) and Wako Diagnostics, (Richmon, USA).

### Immunohistochemistry

Liver tissue was fixed for 24 h in 4% paraformaldehyde (PFA) by immersion. Then, liver samples were cryoprotected overnight in 30% sucrose 0.1 M phosphate buffer, frozen at − 80 °C, and sectioned at 20 μm using a Leica cryostat (Leica, Wetzlar, Germany). Oil Red O (Sigma-Aldrich, St. Louis, USA) staining was performed according to standard procedures and counterstained with hematoxylin-eosin (H-E, Thermo Fisher Scientific, MA, USA). Briefly, liver sections were washed with running tap water for 10 min, rinsed with 60% isopropanol, and stained with Oil Red O mixed with 60% isopropanol for 15 min. The sections were then rinsed with 60% isopropanol, and nuclei were stained with H-E, rinsed in tap water, and mounted in coverslips with aqueous mounting medium. Images were captured using a light microscope (Zeiss microscope; Carl Zeiss Microimaging, GmbH, Oberkochen, Germany) at × 40 magnification from five different fields. Positive areas of Oil Red O staining, corresponding to red droplets, were calculated using a color differentiation system and the result is expressed as the total area of the image using Image J software (U. S. National Institutes of Health, Bethesda, MD, USA).

### Western blot analysis

Protein extracts were prepared from frontal cortex cerebral tissue by mechanic homogenization in ice-cold lysis buffer NP-40 (50 mM Tris-base pH 7.4, 150 mM NaCl, 0.5% Nonidet P-40, 1 mM EDTA) containing a mixture of protease and phosphatase inhibitors (Roche Applied Science, Basel, Switzerland) and centrifuged for 15 min at 14000 rpm at 4 °C. Supernatants were collected, and the total protein concentrations were estimated by BCA assay (Pierce BCA Protein Assay Kit, Thermo Fisher, Waltham, MA, USA). Twenty micrograms from each sample were loaded in a precast 4–20% Tris-Glycine gels (Thermo Fisher Scientific, MA, USA) and transferred to polyvinylidene fluoride (PVDF) membranes (Bio-Rad, CA, USA). Then, membranes were blocked and incubated with the corresponding primary antibody: mouse monoclonal anti-Glial fibrillary acidic protein (GFAP, 1:2500; Sigma-Aldrich, G3893; St. Louis, USA); rabbit polyclonal anti-Ionized calcium-binding adaptor molecule 1 (Iba1, 1:1000; Wako Diagnostics, 016–20,001; Richmond, USA), mouse monoclonal anti-complex V β subunit (CxVβ) (1:1000; Abcam, ab14730; Cambridge, UK), rabbit polyclonal anti-peroxisome proliferator-activated receptor γ co-activator 1α (PGC-1α; 1:200; Santa Cruz Biotechnologies, sc13067; CA, USA), rabbit polyclonal anti-UCP-2 (1:1000; Abcam, ab203244; Cambridge, UK) and mouse monoclonal anti-β-actin (1:40000, Abcam ab49900, Cambridge, UK) to monitor protein loading control. Secondary horseradish peroxidase-conjugated goat anti-mouse (1:5000; Bio-Rad, 170–6516; CA, USA) and goat anti-rabbit (1:5000; Thermo Fisher Scientific, A16110; MA, USA) were used. Immunoreactive bands were detected using an enhanced chemiluminescence reagent (ECL Clarity, Bio-Rad, CA, USA) using the ImageQuant TL Image Analysis system version 7.0 (LAS 4000, GE Healthcare, Chicago, IL, USA). Densitometric quantification was carried out with Image Studio Lite 5.0 software (Li-COR Biosciences, NE, USA). Protein bands were normalized to β-actin levels and expressed as a percentage of the control group.

### Data analysis

Immunohistochemistry images were minimally processed in a uniform matter across treatment groups and were analyzed using ImageJ software (NIH, Bethesda, MD, USA). Results are presented in scatter plots with bars. Statistical analysis was carried out using GraphPad Prism 6.01 software (La Jolla, CA, USA) and IBM SPSS Statistics Version 21.0. (Armonk, NY, USA). Grubbs outlier filter was used for all data and the Shapiro-Wilk normality test was carried out to check normality. When results met normality criteria one-way ANOVA and Levene’s test to analyze homoscedasticity were performed in order to choose suitable post hoc analysis (Tukey’s or Games-Howell’s tests) to determine individual differences. When results did not meet normality criteria Kruskal-Wallis ANOVA was used. For experiments showing weight and glucose curves over time with all different conditions, a mixed ANOVA analysis was carried out and sphericity was checked with Mauchly’s test. When sphericity was violated (weight and glucose results), a multivariate ANOVA analysis was conducted. When results did not meet homogeneity criteria, the Games-Howell post hoc test was carried out. A *p* value equal to 0.05 or less was considered statistically significant.

## Results

### BBB megalin deletion protects to HFD-induced obesity

Megalin deletion in brain endothelial cells was previously shown to be a novel mechanism promoting obesity [10]. Additionally, it is known that brain megalin deletion activates obesity-induced neuropathological mechanisms similar to those found in AD models [9, 10]. On the grounds of the observed hypermetabolic state and weight loss in AD patients even when they are under HFD intake, we wondered how HFD challenge could affect BBB megalin deletion mouse model weight and glucose metabolism. To address this question, EMD and wt animals were fed with HFD and NCD for 4 months. Figure 1a shows weight gain in the grouped animals during this time. EMD mice fed with NCD exhibited higher weight gain compared to wt animals (Fig. 1a), equivalent to the previously reported results [10]. HFD administration induced a significant weight gain in both wt and EMD mice, but such an increase was much more representative in wt group (Fig. 1a). Although fat weight was increased in both wt and EMD mice groups 14 weeks after HFD diet administration (Fig. 1b), only wt mice showed significant fat weight gain compared to those mice fed with NCD (*P* < 0.01; Fig. 1b). The increase in fat mass in EMD mice was found 28.6% lower than that observed in wt mice (Fig. 1b). These results suggest that EMD deficiency may protect against diet-induced adiposity.

We next investigated whether fat mass gains in mice under HFD challenge would lead to improved glucose handling and insulin sensitivity. Indeed, glucose tolerance test 14 weeks after HFD administration revealed a significant protection to glucose intolerance in EMD mice compared to wt group (Fig. 1c) as HFD administration did not change the resulting area under the glucose curve (AUC-GTT) in EMD mice as occurred in wt mice (*P* < 0.05; Fig. 1d). Similar glucose disposal rates upon insulin administration were found between groups (Fig. 1e, f). Together, these results indicate BBB megalin deletion may exert a protective effect on the HFD-induced obesity phenotype.

### HFD-induced lipid dyshomeostasis is attenuated in EMD mice

**Fig. 2:**
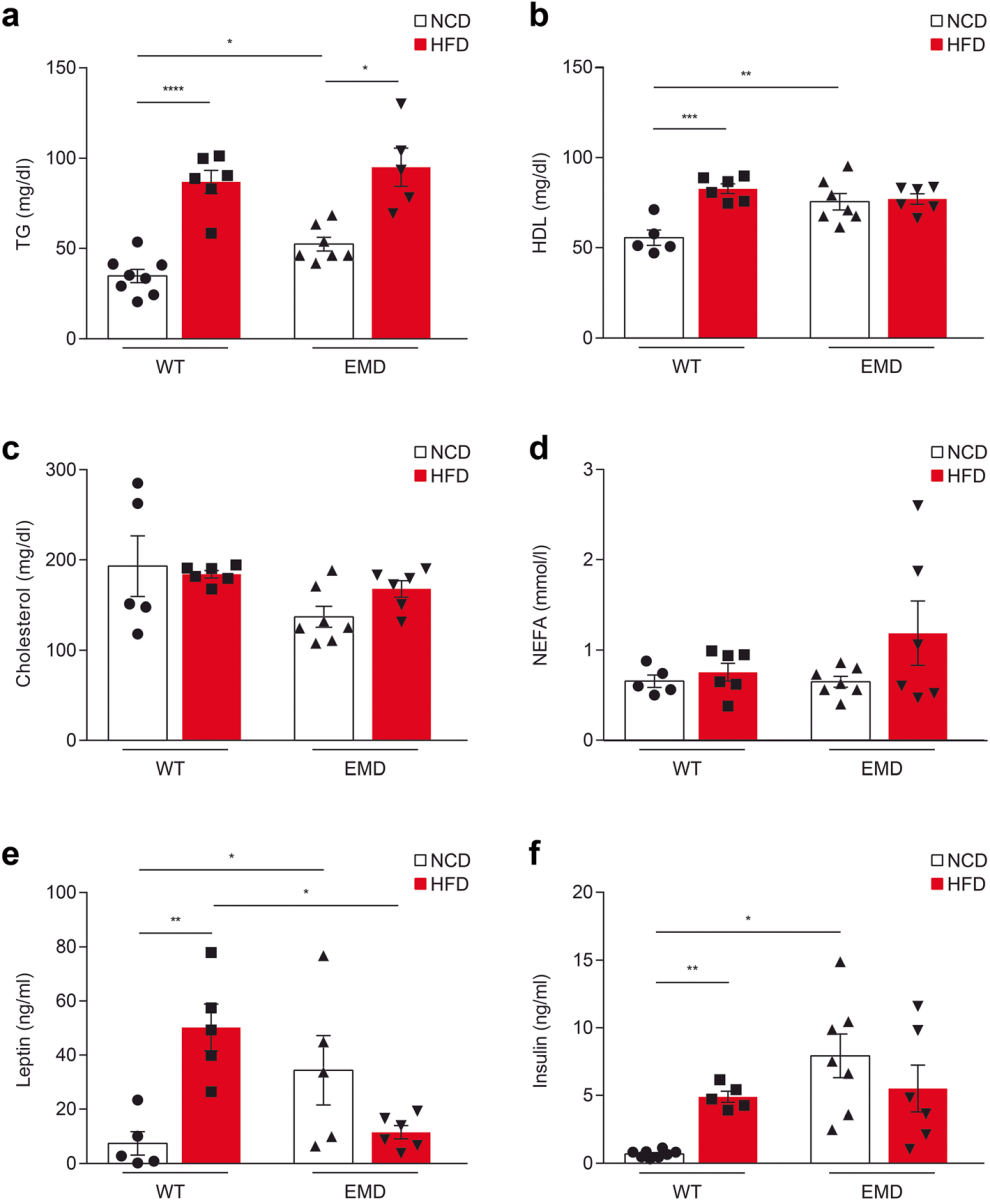
Serum metabolic parameters in wt and EMD mice under NCD and HFD for 14 weeks. (**a–f**) **a** Triglycerides (TG), **b** high-density lipoprotein (HDL), **c** cholesterol, **d** non-esterified *fatty acid* (NEFA), **e** leptin, and **f** insulin tests in wt and EMD mice fed with NCD or HFD for 14 weeks. All data are presented as the mean ± SEM. Statistical significance was based on one-way ANOVA followed by Games-Howell’s (**a**, **c**, **d**, and **f**) or Tukey’s (**b** and **e**) post hoc test. *P < 0.05, **P < 0.01, ***P < 0.001, ****P < 0.0001

Lipid homeostasis in serum was also evaluated. HFD administration resulted in increased serum triglyceride levels in both, wt (*P* < 0.0001; Fig. 2a) and EMD (*P* < 0.05; Fig. 2a) mice compared to mice fed with NCD. However, whereas high-density lipoprotein (HDL) levels in wt mice were found increased upon HFD administration compared to NCD-fed animals (*P* < 0.001; Fig. 2b), no effect on these levels was found in HFD-fed EMD mice. (Fig. 2b). Surprisingly, serum cholesterol levels remained unchanged in EMD mice compared to wt mice under NCD and as well as upon HFD administration (Fig. 2c). No differences were found in non-esterified *fatty acid* (NEFA) among the genotypes and diets (Fig. 2d).

Leptin and insulin levels in serum were found higher in EMD mice compared to wt mice upon NCD administration according to previous data (Leptin = *P* < 0.05; Fig. 2e; Insulin = *P* < 0.05; Fig. 2f). After 14 weeks of HFD, wt mice displayed a significant increase in serum leptin levels (*P* < 0.01; Fig. 2e), whereas HFD challenge did not change serum leptin levels in EMD mice (Fig. 2e). Serum insulin levels were also found increased in wt upon HFD (*P* < 0.01; Fig. 2f) but in EMD group, insulin levels remained unchanged after 14 weeks HFD feeding (Fig. 2f).

### EMD mice display reduced liver damage upon HFD administration

**Fig. 3:**
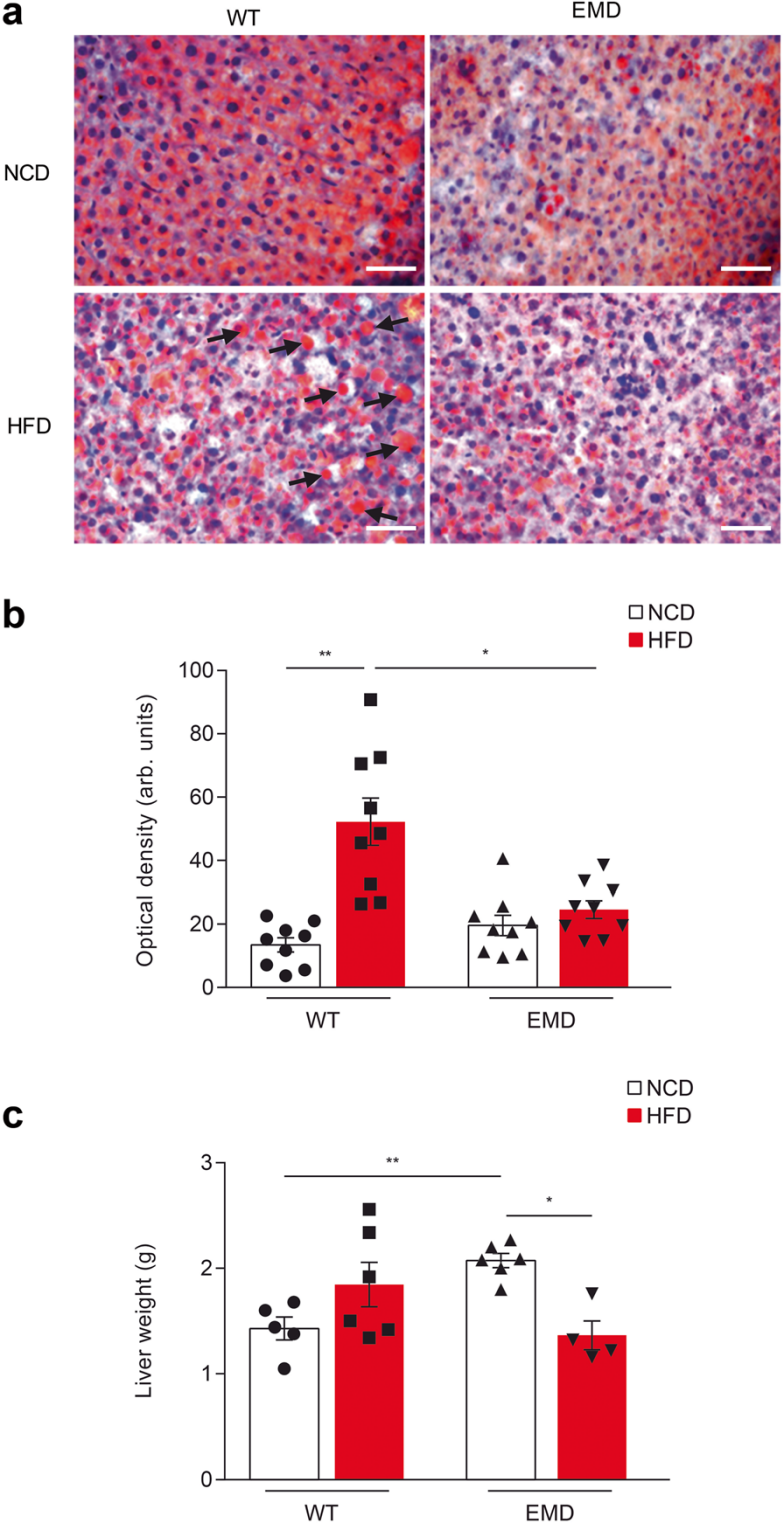
EMD mice are protected against HFD-induced hepatic steatosis. **a** Representative images of Oil Red O and H&E co-stained liver sections from NCD and HFD-fed wt and EMD mice. Arrows indicate lipidic stained droplets. Scale bar: 50 μm. **b** Scatter plots with bars showing the quantification of intracellular fat drops followed by Oil Red O staining. **c** Liver weight in NCD or HFD-fed mice at week 20. All data are presented as the mean ± SEM. Statistical significance was based on one-way ANOVA followed by Games-Howell’s post hoc test. *P < 0.05, **P < 0.01

Linked to lipid homeostasis is the liver health and lipid dyshomeostasis induce liver lipid accumulation. Therefore, we next analyzed lipid accumulation in mice liver sections from HFD-fed mice compared to mice fed with NCD. H&E and Oil Red O-co-staining showed an increased number of red droplets in wt but not in EMD mice upon HFD challenge indicating liver lipid accumulation and hepatic steatosis (Fig. 3a). Oil Red O histological analysis of liver sections showed a significant increase of lipid infiltration in HFD-fed wt mice compared to NCD-fed animals (*P* < 0.01; Fig. 3b). In addition, after 14 weeks of HFD feeding, EMD mice exhibited a significant reduction in liver weight (*P* < 0.05) not observed in wt mice (Fig. 3c).

### HFD challenge does not affect the PGC1-1α and UCP2 brain cortex levels in EMD mice

**Fig. 4:**
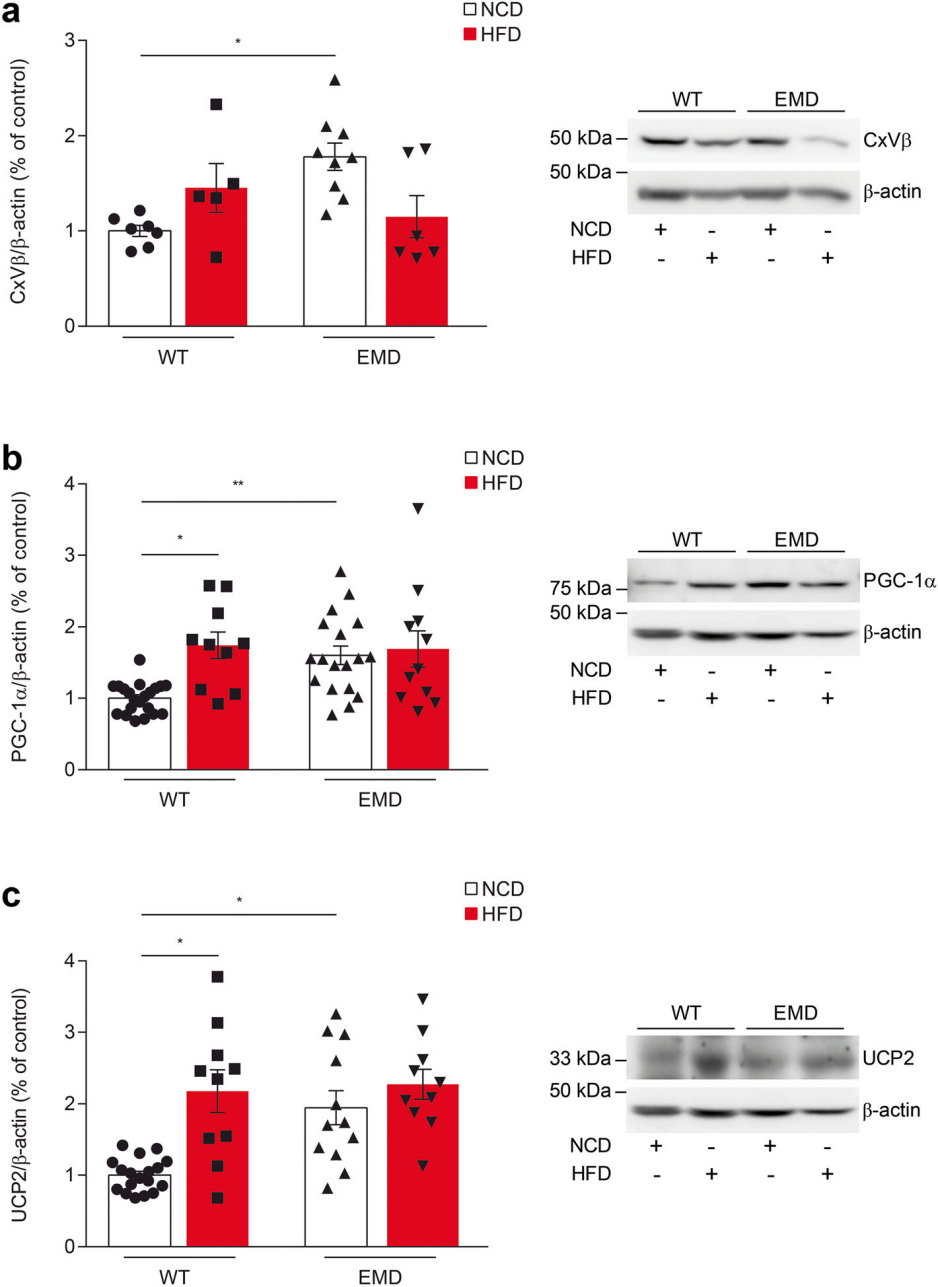
HFD challenge does not affect the PGC1-1α and UCP2 cortex levels in EMD mice. (**a–c) a** Protein levels of Complex V-β subunit (CxVβ), **b** PGC-1α, and **c** UCP-2 were significantly higher in EMD mice cortical samples compared to samples from the same brain area in wt animals under NCD. HFD administration increased **b** PGC-1α, and **c** UCP-2 protein levels in wt mice but not in EMD mice. Scatter plots with bars represent the quantification of protein expression in each animal group, and representative western blots are shown (right panels). All data represent the mean of at least 3 independent experiments ±SEM. Statistical significance was based on the Kruskal-Wallis ANOVA test (**a**) or one-way ANOVA (**b** and **c**) followed by Games-Howell’s post hoc test (**b** and **c**). *P < 0.05, **P < 0.01

We previously demonstrated that mitochondrial mass was increased in brains from EMD mice compared to wt animals [10]. It is known that HFD induces an increase in mitochondrial mass [28]; therefore, we wondered how HFD administration could affect mitochondrial mass levels in the frontal cerebral cortex of EMD mice. To this end, we estimated the amount of total mitochondrial mass in this brain area by analyzing the levels of the structural mitochondrial protein complex V, β subunit (CxVβ) by immunoblotting (Fig. 4a). The mitochondrial mass analysis confirmed the previously reported results showing that upon NCD administration, brain cortical samples from EMD mice displayed significant elevated mitochondrial mass levels compared to wt animals (*P* < 0.01; Fig. 4a,). Upon HFD administration, mitochondrial mass levels remained unchanged. Then, we analyzed PGC-1α levels as this protein is the master regulator of mitochondrial biogenesis in order to verify whether these protein levels could be related to the observed changes in mitochondrial mass upon HFD administration. PGC-1α was found significantly increased in EMD mice frontal cerebral cortex samples compared to samples from the same brain area in wt animals (*P* < 0.01; Fig. 4b). HFD administration induced an increase in PGC-1α protein levels in wt mice compared with animals from the same group fed with NCD (*P* < 0.05; Fig. 4b). However, the HFD challenge did not alter PGC-1α protein levels on EMD mice (Fig. 4b). Apart from the mitochondrial biogenesis, PGC-1α regulates uncoupling protein-2 (UCP2), a thermogenic protein involved in the regulation of energy metabolism. EMD mice fed with NCD showed increased UCP2 levels compared to wt mice in agreement with the highly observed PGC-1α levels (*P* < 0.05; Fig. 4c). HFD administration to wt mice reported an increase in UCP2 levels (*P* < 0.05; Fig. 4c) reaching similar values to those observed in NCD treated EMD mice. No differences in UCP2 levels were observed in EMD mice, indicating this pathway could be already activated (Fig. 4c).

### HFD challenge does not affect glial activation in EMD mice

**Fig. 5:**
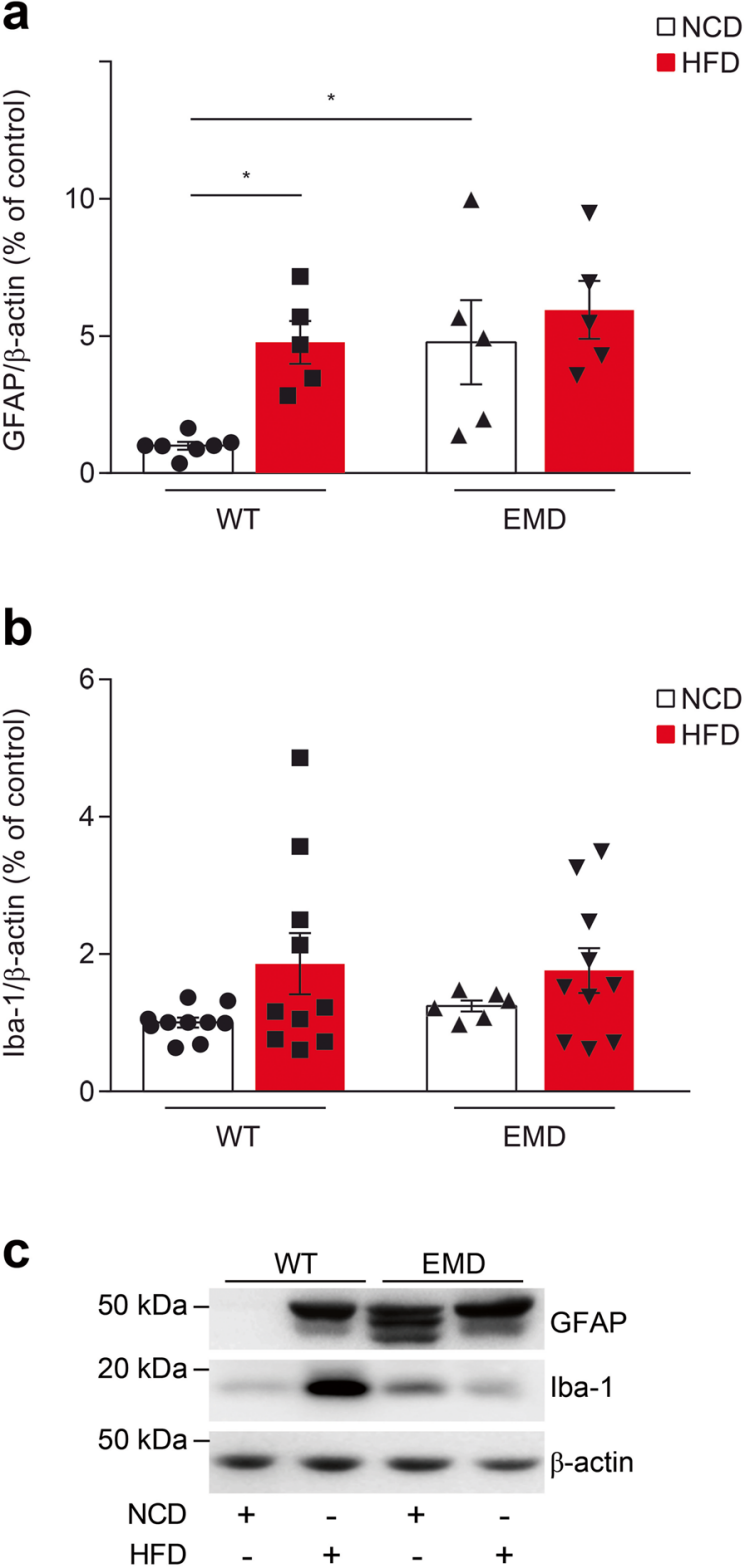
Glial activation markers in wt and EMD mice under NCD and HFD for 14 weeks. **(a–c) a** Protein levels of GFAP, and **b** Iba1 were significantly higher in HFD-fed wt mice compared to NCD fed animals. Upon NCD, GFAP expression was also significantly increased in EMD mice compared to wt mice. **a**, **b** Scatter plots with bars represent protein levels quantification in each animal group. **c** Representative western blots are shown. All data are presented as the mean ± SEM. Statistical significance was based on one-way ANOVA test followed by Games-Howell’s post hoc test (b and c). *P < 0.05

It is known that diet-induced obesity triggers glial activation. A recent study reported a link between mitochondrial dynamics and inflammation, showing that HFD induced an increase in UCP2 expression that mediates neuroinflammation [29]. We then tested astrocyte and microglial inflammatory markers in frontal cerebral cortex from wt and EMD mice upon HFD challenge. Consistent with our previous work [10], we found significant higher GFAP levels, in the cerebral cortex of NCD-fed EMD mice compared to wt mice (*P* < 0.05; Fig. 5a, c) indicating astrogliosis. Upon HFD administration we also found significantly increased GFAP levels in the cerebral cortex of wt mice, compared to NCD-fed animals (*P* < 0.05; Fig. 5a, c) but no changes upon HFD administration were found in EMD mice. The analysis of Iba-1 levels did not report significant differences between mice groups (Fig. 5b, c) although a trend of increased levels was found upon HFD administration in both of them.

## Discussion

Endocytic multiligand receptor megalin internalizes leptin, a hormone modulator of body weight through the balance of food intake and energy expenditure [1]. Here, we show that the HFD-induced obesity phenotype is attenuated in EMD mice compared to wt-fed animals. We found that HFD-fed EMD mice developed reduced body weight gain and improved glucose tolerance. About fat body composition, several interesting observations were observed. High fat diet-fed wt mice demonstrated hyperinsulinemia and hyperleptinemia as well as elevated circulating triglyceride levels, and hepatic steatosis in comparison with control diet-fed mice, consistent with previous studies [30]. However, over the 14 weeks HFD-treatment period, EMD mice exhibited less overweight, and fat gain compared with wt mice, and contrary to expected, leptin and insulin levels did not change. Also, HFD-fed EMD mice did not show hepatic steatosis. Upregulation of PGC-1α and thermogenic protein UCP2 was observed in the brain cortex from HFD-fed wt mice. Such increases were not apparent in EMD mice as both PGC-1α and UCP2 levels were found already increased with normal chow diet. This result is consistent with the previously reported increased mitochondrial mass detected in EMD mice compared to wt as PGC-1α is a master regulator of mitochondrial biogenesis. Our results suggest BBB endothelial megalin deletion protects HFD-induced obesity in mice, insulin resistance and hepatic steatosis through brain endocrine system communications.

Our present study agrees with previous findings that identify LRP-6 as bodyweight and glucose metabolism regulator [31]. In that study, authors reported that LRP6^+/−^ mice on high-fat diet are protected against diet-induced obesity. We previously demonstrated that the silencing of megalin in the mouse brain endothelium was sufficient to increase the weight gain and adiposity triggering hyperleptinemia, hyperinsulinemia, increased triglyceride blood levels, and impaired glucose tolerance [10]. Additionally, we provided evidences supporting megalin as physiological energy balance regulator in agreement with other works [32–34].

Megalin knockout mice manifest abnormalities in the development of brain and other tissues, including lung and kidney [35]. This phenotype is consistent with a role of megalin as endocytic receptor that mediates the cellular uptake of essential nutrients, possibly lipoproteins-derived cholesterol. A series of preceding biochemical and experimental studies have provided compelling evidence showing that megalin plays an important role in modulating protein and lipids transport [4]. Indeed, several studies revealed that megalin can also act as a receptor to transport leptin in the renal epithelium [6, 36]. Additionally, in a previous work, we showed that leptin entry into the brain occurs through its binding to megalin and the effects of blocking megalin expression indicated that leptin needs megalin to exert its function in the brain [5]. Then, food intake regulation and subsequent energy balance depend on the efficiency of leptin delivery in CNS [37]. There are not consistent results regarding the effects of high-fat diet administration compared to standard diets on triglyceride levels. Guo et al. showed mice fed for 7 weeks with obesogenic diets (60% high fat diet enriched) exhibited lower serum triglyceride levels compared with normal chow diet [38]. Contrarily, in a previous work, we demonstrated western-style high fat diet in rats fed during 1 and 3 months increased lipid profile levels including triglycerides and HDL compared to rats fed with a standard diet [39]. We confirm here the obesity phenotype in EMD mice as they show increased triglyceride levels along with hyperleptinemia and hyperinsulinemia. We did not find changes in NEFA levels between groups and diets.

Although cholesterol levels between EMD mice and wt were not found different, other blood metabolic parameters showed typical obese phenotype as increased triglyceride levels, increased insulin and increased leptin levels. Surprisingly, increased HDL levels were also found to increase in EMD mice compared to wt. This may account for undetectable differences in cholesterol levels between EMD and wt mice. Upon HFD administration wt mice showed increased blood levels of triglycerides, insulin and leptin, and also HDL, while EMD mice only showed enhanced triglyceride levels but did not show changes in the already increased insulin, leptin, and HDL blood levels. Since megalin mediates HDL endocytosis [40], the lack of this receptor in EMD mice may impair HDL brain uptake resulting in the observed high circulating HDL levels, regardless of feeding. Our results here agree with those from Dietrich et al., as we found wt mice exhibited increased triglyceride and HDL levels after HFD administration. As was demonstrated in previous works, we may speculate such controversy comes from the timing of HFD feeding, animal strain and/or diet composition [41, 42]. Under NCD, EMD mice showed significantly increased serum HDL and triglyceride levels compared with WT mice.

Diet-induced obesity is a well-known model of hyperleptinemia and central leptin resistance [43, 44]. In rodents, high-fat intake may be associated with increased serum leptin and obesity, and these leptin levels are related to the body lipid content. In the present study, EMD mice showed no changes in serum leptin concentrations after HFD. As EMD mice showed lower fat gain with HFD and leptin is secreted in proportion to fat stores [45], it is possible that EMD mice are more efficient maintaining leptin sensitivity compared to wt mice throughout the course of the HFD administration period even to show leptin levels reduction.

Diet-induced obesity also predisposes individuals to insulin resistance [46]. Elevations in circulating insulin were evident in both mouse models of obesity used in our study: genetic (EMD mice) and diet-induced obesity model (HFD-fed wt mice). The present findings are important because hyperinsulinemia is a risk factor for many, if not all, symptoms used to sort out the metabolic syndrome and elevated insulin levels have been suggested to be a causal factor for obesity [47, 48].

An increase in liver fat content has been shown to predict insulin resistance. It is generally thought that hepatic steatosis is developed via peripheral mechanisms associated with obesity [49]. However, in our present study, we show that EMD mice did not exhibit liver steatosis. Some evidences indicate that experimental exercise can prevent steatosis in HFD-induced obesity [50, 51]. Here we report increased anxiety behavior in NCD-fed EMD mice. This was demonstrated in experiments carried out using the elevated plus maze. EMD showed increased number of entries and time spent in the open-arms. These results indicated that EMD mice are more prone to physical activity. This finding connecting increased anxiety in EMD mice and greater physical activity may likely contribute to attenuate their liver steatosis.

Mitochondrial bioenergetics may be influenced by insulin signaling [52]. At least in some tissues, impaired mitochondrial function causes insulin resistance [53]. Our previous work showed endothelial BBB megalin deletion was associated with deficient mitochondrial complex I in the brain cortex but increased mitochondrial mass [10]. Then, we proposed that such mitochondrial mass increase in EMD mice could be a consequence of higher mitochondrial biogenesis. Indeed, here, we verified PGC-1α levels were increased in frontal cerebral cortex from EMD mice. PGC-1α is expressed in the brain, including the cerebral cortex, and hippocampus [54, 55] and this protein is the master regulator for mitochondrial biogenesis. Therefore, cells may compensate for the energetic deficit due to reduced complex I levels by increasing the mitochondrial biogenesis. HFD administration increased the PGC-1α levels in wt mice and therefore the mitochondrial biogenesis was found upregulated. However, HFD did not affect the mitochondrial biogenesis already increased in EMD mice. Although this is controversial, several previous works demonstrated similar features showing increased mitochondrial content in skeletal muscle after HFD administration [28, 56, 57] suggesting such increase in the mitochondrial content could be beneficial for improving insulin resistance at the beginning of HFD [58]. In addition, we also found increased UCP2 levels in EMD mice compared to wt animals, and HFD induced an increase of these protein levels in wt mice but did not affect the already increased levels in EMD mice. PGC-1α regulates UCP2 protein expression and this protein plays an important role in the regulation of energy metabolism restoring glucose intolerance and insulin resistance [59, 60]. Regarding glucose metabolism, PGC-1α has been associated with glucose intolerance and insulin resistance as has been demonstrated using PGC-1α knockout mice [61]. For example, loss of function or lower expression levels of PGC-1α has been associated with increased risk of type 2 diabetes [62]. As suggested by Summermatter et al., PGC-1α could be involved in glucose refueling and body lactate homeostasis [63]. Mitochondrial uncoupling proteins as UCP2 could also regulate glucose homeostasis and lipid metabolism [64]. UCPs increases their neuronal expression induced by metabolic changes and several works link UCP2 levels in the brain and systemic metabolic abnormalities. For example, UCP2 was found to increase in cerebral cortex mitochondria after exercise [65]. UCP2 is involved in central autonomic, endocrine, and metabolic regulation and is thus associated with cognition, mood, and behavior [66, 67]. UCP2 in the ventromedial nucleus restores glucose tolerance and regulates insulin sensitivity mediated by glucose-excited neurons, which is important for the physiological control of systemic glucose metabolism [68]. We may propose that metabolic changes in EMD mice induce elevated PGC-1α and UCP2 brain cortical expression and we may hypothesize such elevated expression could be considered a potential prevention mechanism against HFD challenge trying to compensate defective mitochondria. This potential prevention mechanism may be extensible against the brain inflammation pathways as EMD mice exposed to HFD did not show evidence of increased glial activation compared with mice from the same group exposed to NCD. This protective effect might be the result of higher energy expenditure, based on the levels of the thermogenic factor PGC-1α that were found significantly increased in the brain cortex from EMD mice compared to wt.

## Conclusion

In summary, EMD mice recapitulate several features found in human dementia as AD, suggesting megalin as a control gate for metabolic homeostasis. We may conclude that the metabolic phenotype of HFD-fed EMD mice may be, at least in part, explained by improved glucose tolerance.

## Data Availability

The datasets supporting the conclusions of this article are included within the article and its additional files.

## Abbreviations

AD: Alzheimer’s disease
BBB: Blood-brain barrier
CNS: Central nervous system
CxVβ: Mitochondrial complex V, β subunit
EMD: Endothelial megalin deficient
HDL: High-density lipoprotein
HFD: High-fat diet
LRP: Low-density lipoprotein receptor-related protein receptor
NCD: Normal chow diet
NEFA: Non-esterified *fatty acid*s
PGC-1α: Transcriptional coactivator peroxisome proliferator-activated receptor α coactivator
TG: Triglyceride
UCP2: Uncoupling protein-2
